# Supplementary material for: Structure of the T. brucei kinetoplastid RNA editing substrate-binding complex core component, RESC5
Source: PLoS One. 2023 Mar 2;18(3):e0282155. doi: 10.1371/journal.pone.0282155 (PMC9980740; doi:10.1371/journal.pone.0282155)
Supplement: S1 Fig — Shown are the fractions obtained from the SEC analyses of RESC5 (fractions 27–29) in the last three protein lanes, see Fig 1A. (PDF) [file pone.0282155.s001.pdf]

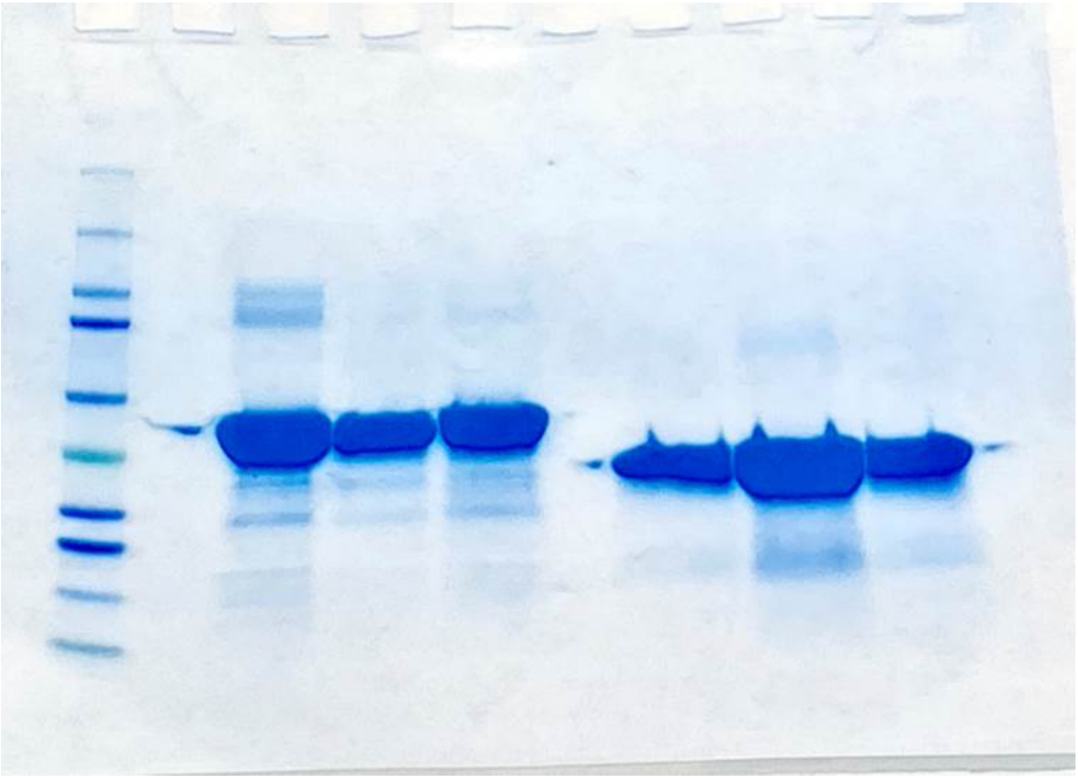

S1 Fig. Uncropped gel shown in Fig. 1A. Shown are the fractions obtained from the SEC analysis of RESC5 (fractions 27-29) in the last three protein lanes, see Fig 1A.
