# Supplementary material for: Structure of the T. brucei kinetoplastid RNA editing substrate-binding complex core component, RESC5
Source: PLoS One. 2023 Mar 2;18(3):e0282155. doi: 10.1371/journal.pone.0282155 (PMC9980740; doi:10.1371/journal.pone.0282155)
Supplement: S2 Fig — A Purification of RESC5 showing SDS PAGE analyses of fractions collected from Cobalt NTA purification. The top labels indicate imidazole concentrations used to elute the given fractions. B Sections of simulated annealing composite omit map calculated in Phenix for the RESC5 structure and contoured at 1σ. (PDF) [file pone.0282155.s002.pdf]

A Purification of RESC5

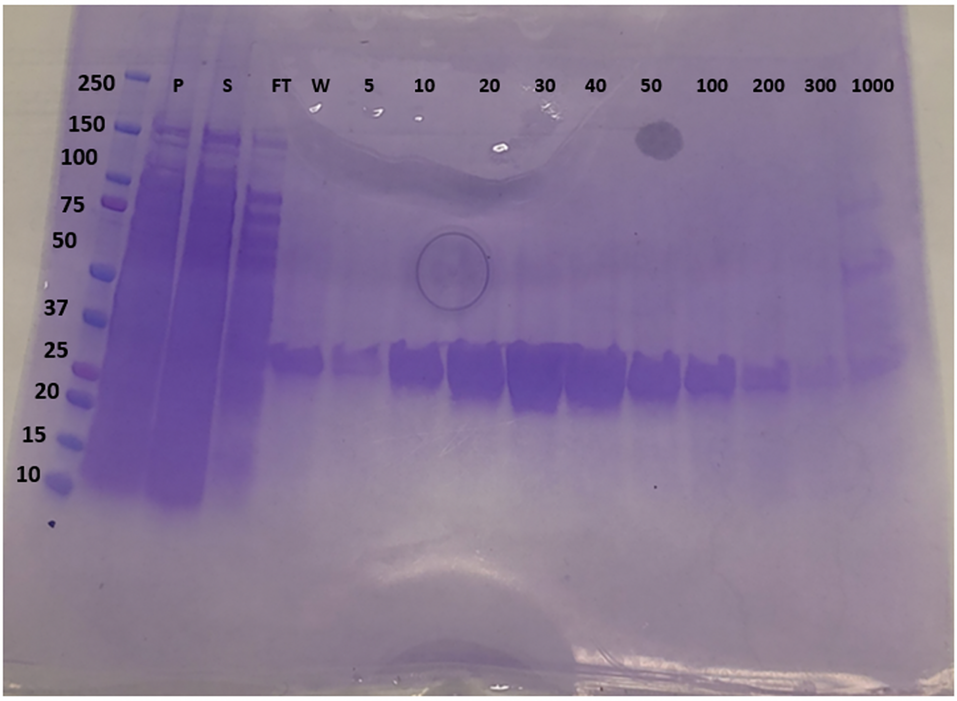

B RESC5 composite omit map

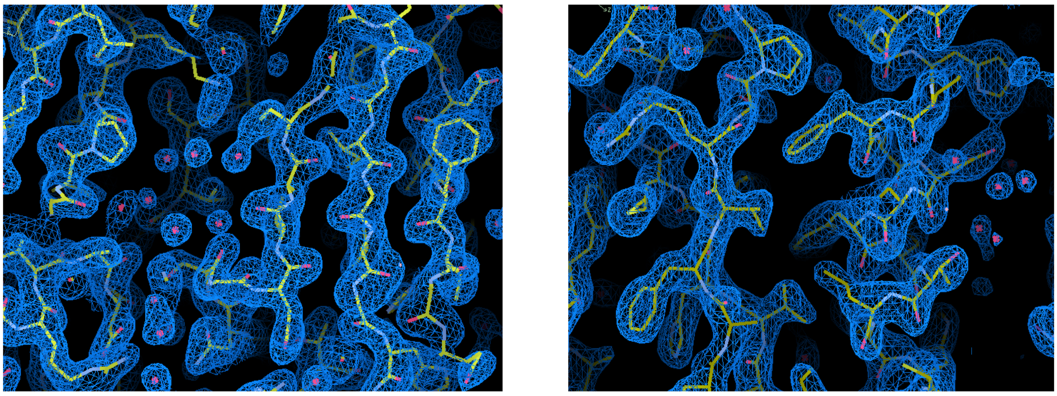

S2 Fig. Purification and composite omit electron density map for RESC5. A Purification of RESC5 showing SDS PAGE analyses of fractions collected from Cobalt NTA purification. The top labels indicate imidazole concentrations used to elute the given fractions. **B** Sections of simulated annealing composite omit map calculated in Phenix for the RESC5 structure and contoured at 1 σ.
