# Supplementary material for: Structure of the T. brucei kinetoplastid RNA editing substrate-binding complex core component, RESC5
Source: PLoS One. 2023 Mar 2;18(3):e0282155. doi: 10.1371/journal.pone.0282155 (PMC9980740; doi:10.1371/journal.pone.0282155)
Supplement: S3 Fig — RESC5 (pink) was superimposed into the IF6 (pdb code: 1G61) structure (cyan) resulting in an rmsd of 3.5 Å for 1170 Cα atoms. (PDF) [file pone.0282155.s003.pdf]

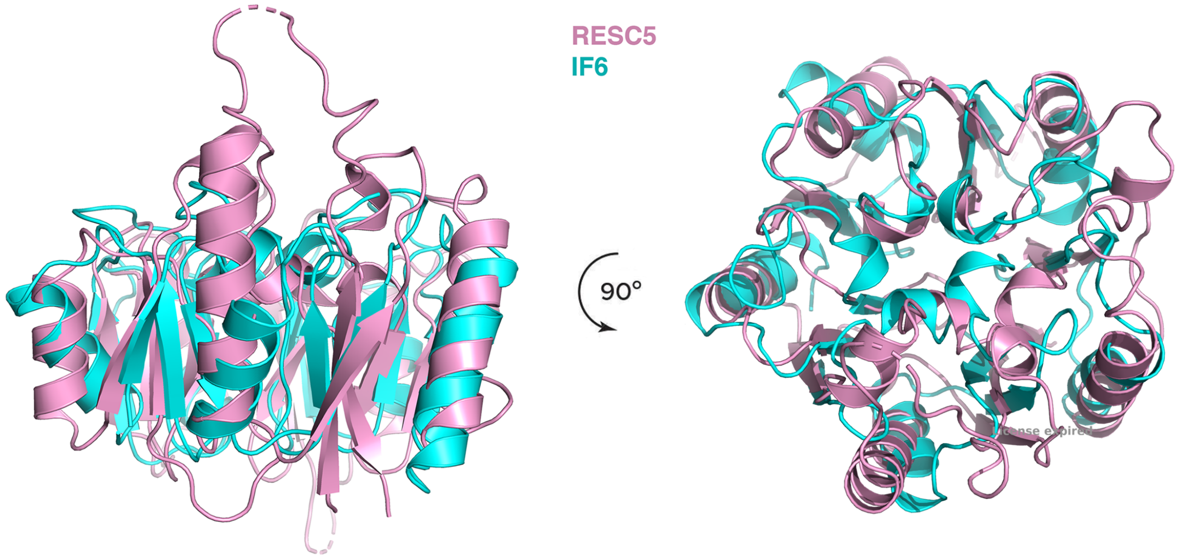

S3 Fig. Overlay of RESC5 onto the *M. jannaschii* IF6. RESC5 (pink) was superimposed into the IF6 (pdb code: 1G61) structure (cyan) resulting in an rmsd of 3.5 Å for 165 Cα atoms. Shown are two views of the overlay related by a 90° rotation.
