# Supplementary material for: Structure of the T. brucei kinetoplastid RNA editing substrate-binding complex core component, RESC5
Source: PLoS One. 2023 Mar 2;18(3):e0282155. doi: 10.1371/journal.pone.0282155 (PMC9980740; doi:10.1371/journal.pone.0282155)
Supplement: S4 Fig — Analysis of putative pockets (right). The only significant pocket (checked in this list) with a druggability score of 312 is that corresponding to the catalytic site in DDAH enzymes. The pocket is shown superimposed on the RESC5 cartoon at the left. (PDF) [file pone.0282155.s004.pdf]

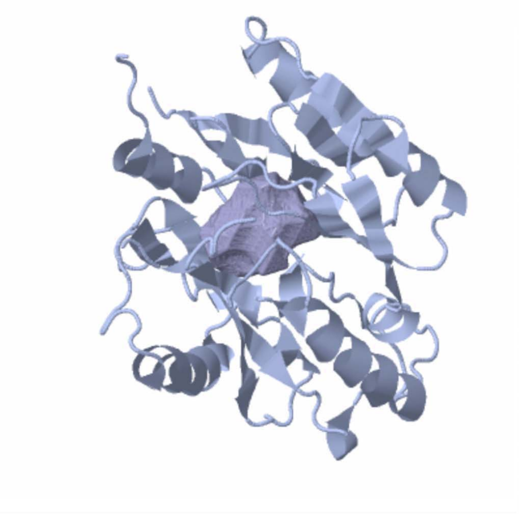

▾ Cavity Results

No.	Pred. Max pKd	Pred. Avg pKd	DrugScore	Druggability	Surface	Residues
1	10.49	6.94	-462.00	Weak	☐	More
2	11.58	6.59	312.00	Medium	☑	More
3	11.53	6.57	38.00	Medium	☐	More
4	10.98	6.38	-331.00	Weak	☐	More
5	9.59	5.91	-517.00	Weak	☐	More
6	9.46	5.86	-754.00	Weak	☐	More
7	8.53	5.54	-666.00	Weak	☐	More
8	8.31	5.47	-1325.00	Weak	☐	More
9	7.07	5.04	-982.00	Weak	☐	More
10	6.97	5.01	-1030.00	Weak	☐	More
11	6.42	4.82	-999.00	Weak	☐	More
12	5.67	4.56	-1343.00	Weak	☐	More

S4 Fig. Assessment of putative pockets in the RESC5 structure by CavityPlus. Analysis of putative pockets (right). The only significant pocket (checked in this list) with a druggability score of 312 is that corresponding to the catalytic site in DDAH enzymes. The pocket is shown superimposed on the RESC5 cartoon at the left.
