# Supplementary material for: Structure of the T. brucei kinetoplastid RNA editing substrate-binding complex core component, RESC5
Source: PLoS One. 2023 Mar 2;18(3):e0282155. doi: 10.1371/journal.pone.0282155 (PMC9980740; doi:10.1371/journal.pone.0282155)
Supplement: S5 Fig — Ribbon diagram showing the overlay of RESC5 (colored pink) with DDAH (colored cyan) and residues subjected to mutation for biochemical assays. Also shown as stick is the location of where the product citrulline binds in the DDAH enzyme. (PDF) [file pone.0282155.s005.pdf]

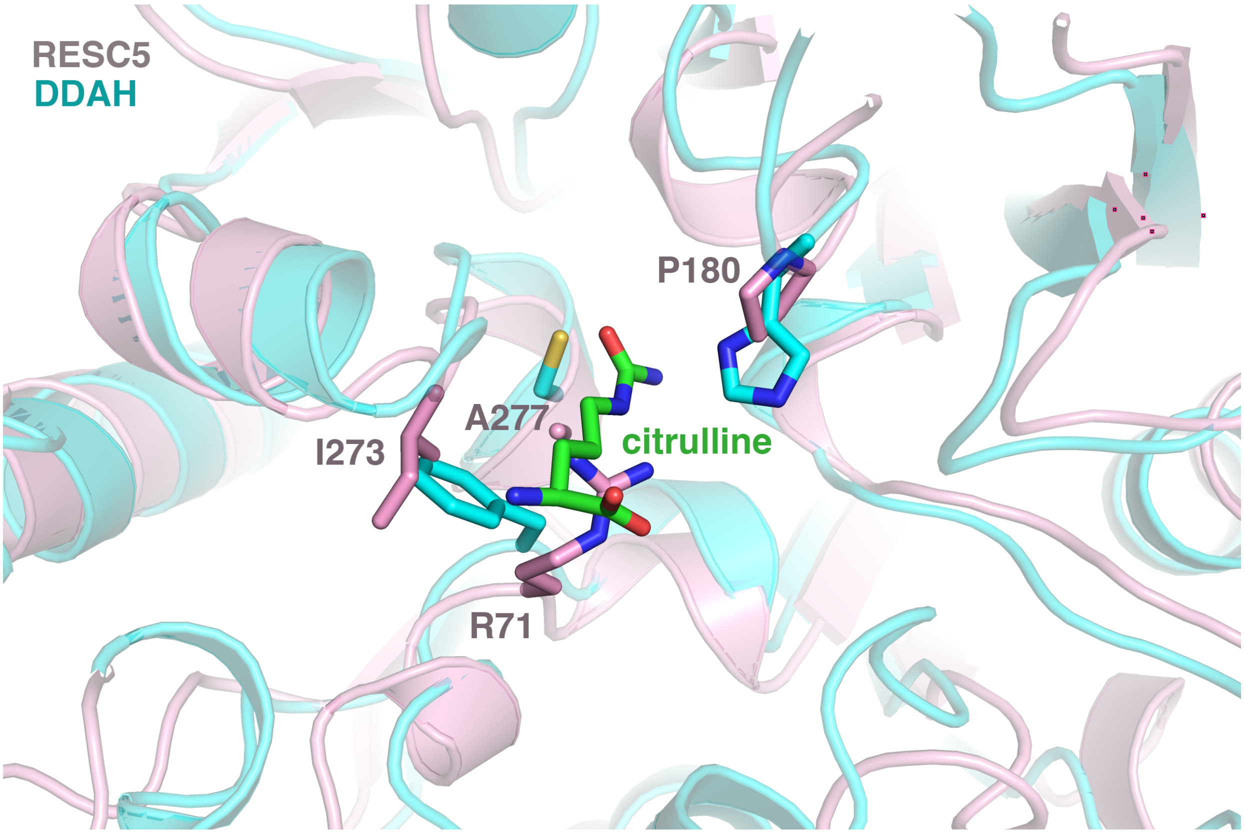

S5 Fig. The RESC5(R71A-P180H-A277C) mutant does not bind DDAH substrate or product. Ribbon diagram showing the overlay of RESC5 (colored pink) with DDAH (colored cyan) and residues subjected to mutation for biochemical assays. Also shown as stick is the location of where the product citrulline binds in the DDAH enzyme.
