# Supplementary material for: Structure of the T. brucei kinetoplastid RNA editing substrate-binding complex core component, RESC5
Source: PLoS One. 2023 Mar 2;18(3):e0282155. doi: 10.1371/journal.pone.0282155 (PMC9980740; doi:10.1371/journal.pone.0282155)
Supplement: S1 File — (PDF) [file pone.0282155.s006.pdf]

Supplemental Information for:

Structure of the T. brucei kinetoplastid RNA editing substrate-binding complex core component RESC5

Maria A. Schumacher*, Emily Cannistraci and Raul Salinas

Department of Biochemistry, Duke University School of Medicine, Box 3711, DUMC Durham, NC 27710, U.S.A.

*To whom correspondence should be addressed: Maria A. Schumacher: maria.schumacher@duke.edu

A Purification of RESC5

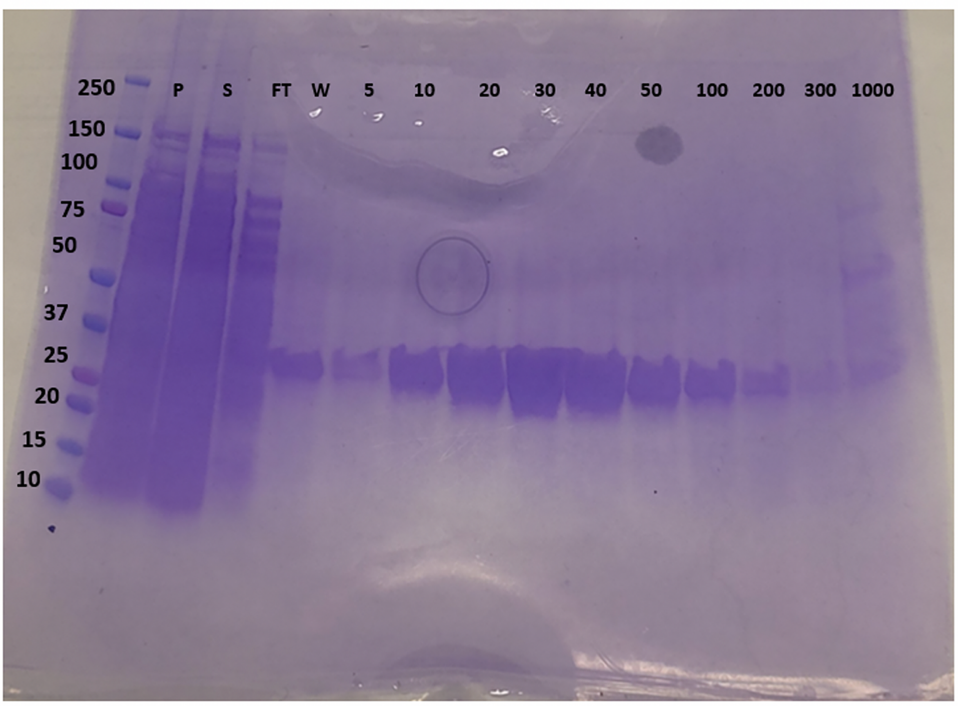

B RESC5 composite omit map

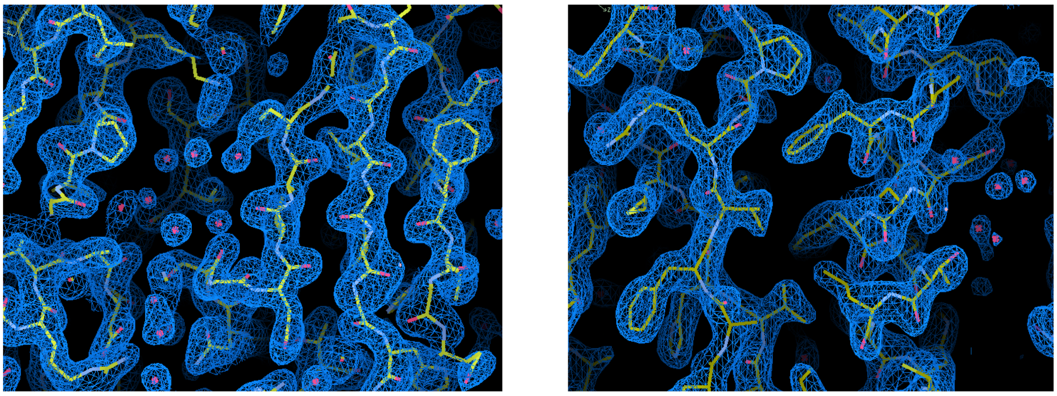

S1 Fig. Purification and composite omit electron density map for RESC5. A Purification of RESC5 showing SDS PAGE analyses of fractions collected from Cobalt NTA purification. The top labels indicate imidazole concentrations used to elute the given fractions. **B** Sections of simulated annealing composite omit map calculated in Phenix for the RESC5 structure and contoured at 1 σ.

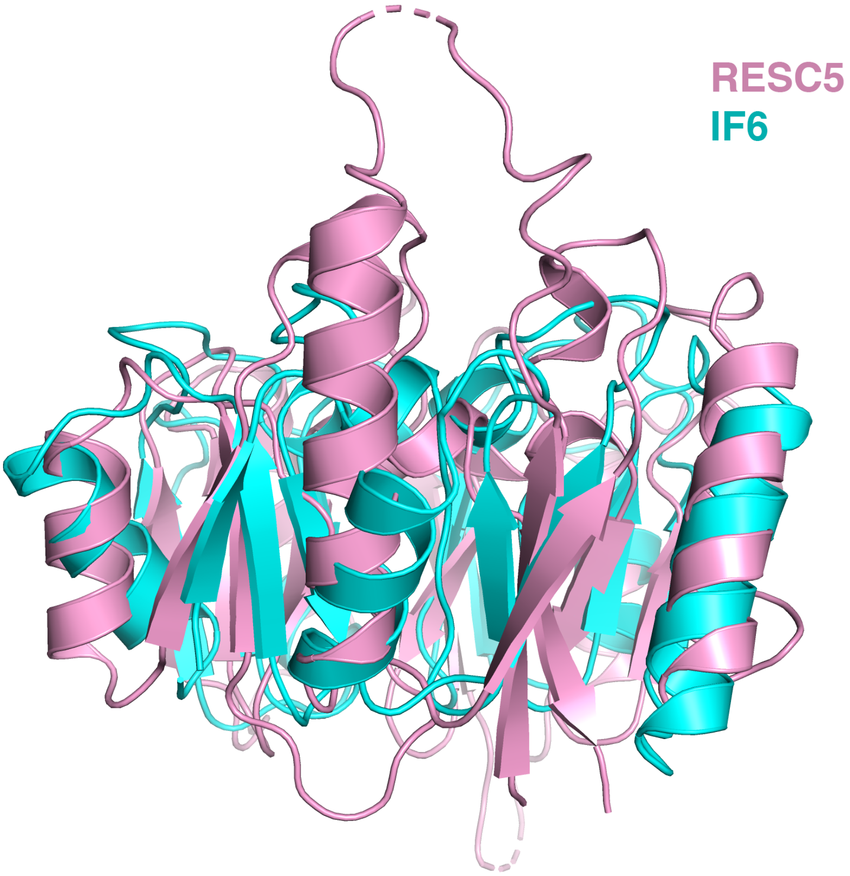

S2 Fig. Overlay of RESC5 onto the *M. jannaschii* IF6. RESC5 (pink) was superimposed into the IF6 (pdb code: 1G61) structure (cyan) resulting in an rmsd of 3.5 Å for 1170 Cα atoms.

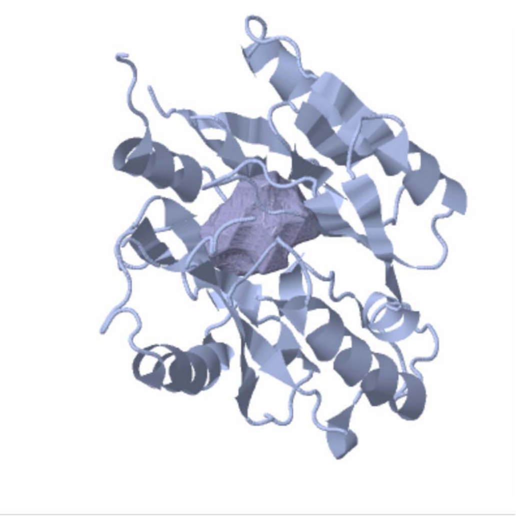

▼ Cavity Results

No.	Pred. Max pKd	Pred. Avg pKd	DrugScore	Druggability	Surface	Residues
1	10.49	6.94	-462.00	Weak	☐	More
2	11.58	6.59	312.00	Medium	☑	More
3	11.53	6.57	38.00	Medium	☐	More
4	10.98	6.38	-331.00	Weak	☐	More
5	9.59	5.91	-517.00	Weak	☐	More
6	9.46	5.86	-754.00	Weak	☐	More
7	8.53	5.54	-666.00	Weak	☐	More
8	8.31	5.47	-1325.00	Weak	☐	More
9	7.07	5.04	-982.00	Weak	☐	More
10	6.97	5.01	-1030.00	Weak	☐	More
11	6.42	4.82	-999.00	Weak	☐	More
12	5.67	4.56	-1343.00	Weak	☐	More

S3 Fig. Assessment of putative pockets in the RESC5 structure by CavityPlus. Analysis of putative pockets (right). The only significant pocket (checked in this list) with a druggability score of 312 is that corresponding to the catalytic site in DDAH enzymes. The pocket is shown superimposed on the RESC5 cartoon at the left.
